# Mechanochemical Formation Mechanism of Alloyed AgBi-Elpasolites

**DOI:** 10.1021/jacs.5c05045

**Published:** 2025-07-03

**Authors:** Huygen J. Jöbsis, Loreta A. Muscarella, Michał Andrzejewski, Nicola P.M. Casati, Eline M. Hutter

**Affiliations:** 1 Department of Chemistry, Utrecht University, Princetonlaan 8, Utrecht 3584 CB, The Netherlands; 4 Swiss Light Source, Paul Scherrer Institute, Forschungstrasse 111, Villigen 5232, Switzerland

## Abstract

Mechanochemical ball mill synthesis is an emerging method for producing complex materials, including alloyed halide elpasolite semiconductors. This solvent-free method offers precise control over chemical composition, enabling fine-tuning of the optical and mechanical properties. However, the formation mechanism of alloyed elpasolites remains unclear. In this work, we elucidate the crystallization kinetics of mechanochemical formation of Cs_2_AgBi_0.5_M_0.5_Br_6_ [M = Sb^3+^, In^3+^, or Fe^3+^] using *in situ* synchrotron X-ray diffraction experiments. We identify the reaction intermediates for the parent composition Cs_2_AgBiBr_6_, and we find that −Bi_0.5_Sb_0.5_– forms via a similar reaction pathway. Alloying with In^3+^ or Fe^3+^, on the other hand, occurs via an additional cation-exchange step. These insights into the mechanochemical formation mechanisms of alloyed AgBi-elpasolites provide guidelines toward rational compositional engineering of complex materials.

## Graphical abstract

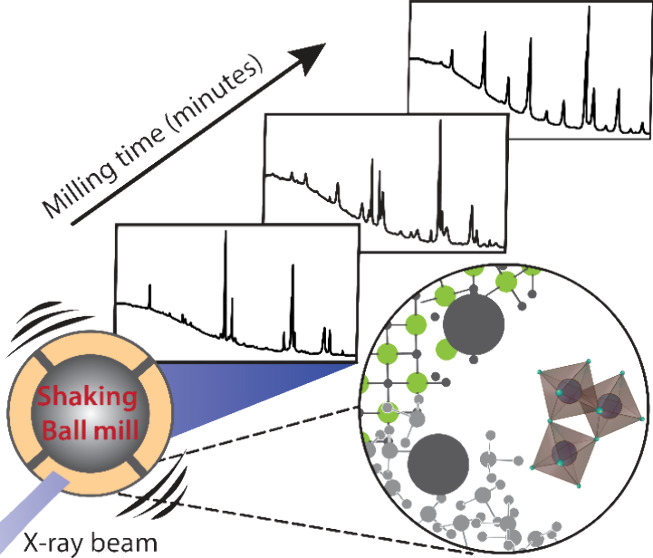

## Introduction

Mechanochemical synthesis methods, such as ball milling, are scalable from the lab scale to industrial levels.^1–4^ In general, mechanochemistry is emerging as a green chemistry method, as it avoids the use of (harmful) solvents.^3–6^ In addition, solvent-free mechanochemical methods are specifically suitable for synthesis of high entropy alloys, i.e., materials comprising five or more elements, as it circumvents solubility issues.^7^ Therefore, mechanochemical synthesis is ideally suited as a screening method toward new, complex materials.^8–10^ We recently found that mechanochemical ball milling is a straightforward solid-state synthesis route to synthesize alloyed Cs_2_AgBi_1–*y*_M_*y*_Br_6_ elpasolites, where *y* can be tuned by varying initial precursor stoichiometry.^11,12^ These halide-elpasolite semiconductor materials show promise for radiation detection, photocatalysis and (indoor) photovoltaics.^13–20^ Compositional engineering of these materials is a promising strategy toward obtaining semiconductors with the desired optical and mechanical properties, based on abundant elements with minimal toxicity. For example, the systematic exchange of the halide from chloride to bromide to iodide gradually decreases the bandgap energy.^21–25^ Furthermore, the bandgap energy of Cs_2_AgBiBr_6_ can be increased upon partly replacing Bi^3+^ with In^3+^ or decreased using Sb^3+^ or Fe^3+^ as a Bi^3+^ substituent.^26–29^ The (partial) substitution of Bi^3+^ with other trivalent metal cations also affects the nature of the bandgap.^30–32^ For instance, both In^3+^ and Fe^3+^ may create direct transitions in Cs_2_AgBiBr_6_, which is in principle favorable for light absorption and emission.^11,26^ Beyond enhancing light absorption for photovoltaic applications,^20,33^ elpasolites have been alloyed with lanthanides^34^ or magnetic centers^35^ to induce luminescent or magnetic properties, respectively. However, the synthesis of alloyed halide elpasolites via established solvent-based routes is largely hampered by the solubility issues of precursors as well as reaction intermediates.

To unlock the full potential of mechanochemical ball mill synthesis to steer the optical properties of AgBi-elpasolites, it is essential to understand the reaction pathways during milling. However, as ball milling involves closed containers, often made of hard and optically not transparent materials, e.g., tungsten carbide, stainless steel, or zirconium oxide, that continuously move, it is not straightforward to probe the reaction mechanism inside the milling jar. Therefore, *ex situ* studies are often performed on the reaction products only,^5,36,37^ while the reaction mechanism and kinetics remain elusive. To tackle this issue, novel reactor designs were recently developed to track reaction conditions,^38^ or to obtain structural or chemical information during the milling reaction.^39–41^

In this work, we use *in situ* powder X-ray diffraction (XRD) ball mill experiments to study the formation mechanism of Cs_2_AgBiBr_6_ and alloyed Cs_2_AgBi_0.5_M_0.5_Br_6_ with M = Sb^3+^, In^3+^, or Fe^3+^. By analyzing the crystalline species during the reaction, we reveal the compound CsBi_2_Br_7_ as a previously unknown intermediate species in the synthesis of Cs_2_AgBiBr_6_, affecting the current understanding of the reaction mechanism. Furthermore, we find that Sb^3+^-alloyed Cs_2_AgBi_0.5_Sb_0.5_Br_6_ forms via a similar reaction pathway. On the other hand, Fe^3+^- and In^3+^-alloying proceeds via an additional reaction step where first the elpasolite phase is formed after which the M-substituents are incorporated.

## Results and Discussion

Powders of Cs_2_AgBi_1–*x*_M_*x*_Br_6_ (M = Sb^3+^, In^3+^, and Fe^3+^) were prepared via ball milling of the precursor powders CsBr, AgBr, BiBr_3_, and MBr_3_ in the desired final stoichiometry. We studied the mechanochemical formation mechanism of AgBi-elpasolites *in situ*, using an in-house designed ball mill setup at the synchrotron radiation facilities at the Swiss Light Source (beamline X04SA-MS, Paul Scherrer Institute).^39^ XRD patterns were collected every 20 s during ball mill synthesis (Figure 1a). Before the ball mill reaction, we ground each reactant manually to ensure similar grain sizes and proper exchange between the inner and outer compartments of the grinding jar (for more experimental details, see Supporting Information).

**Figure 1:**
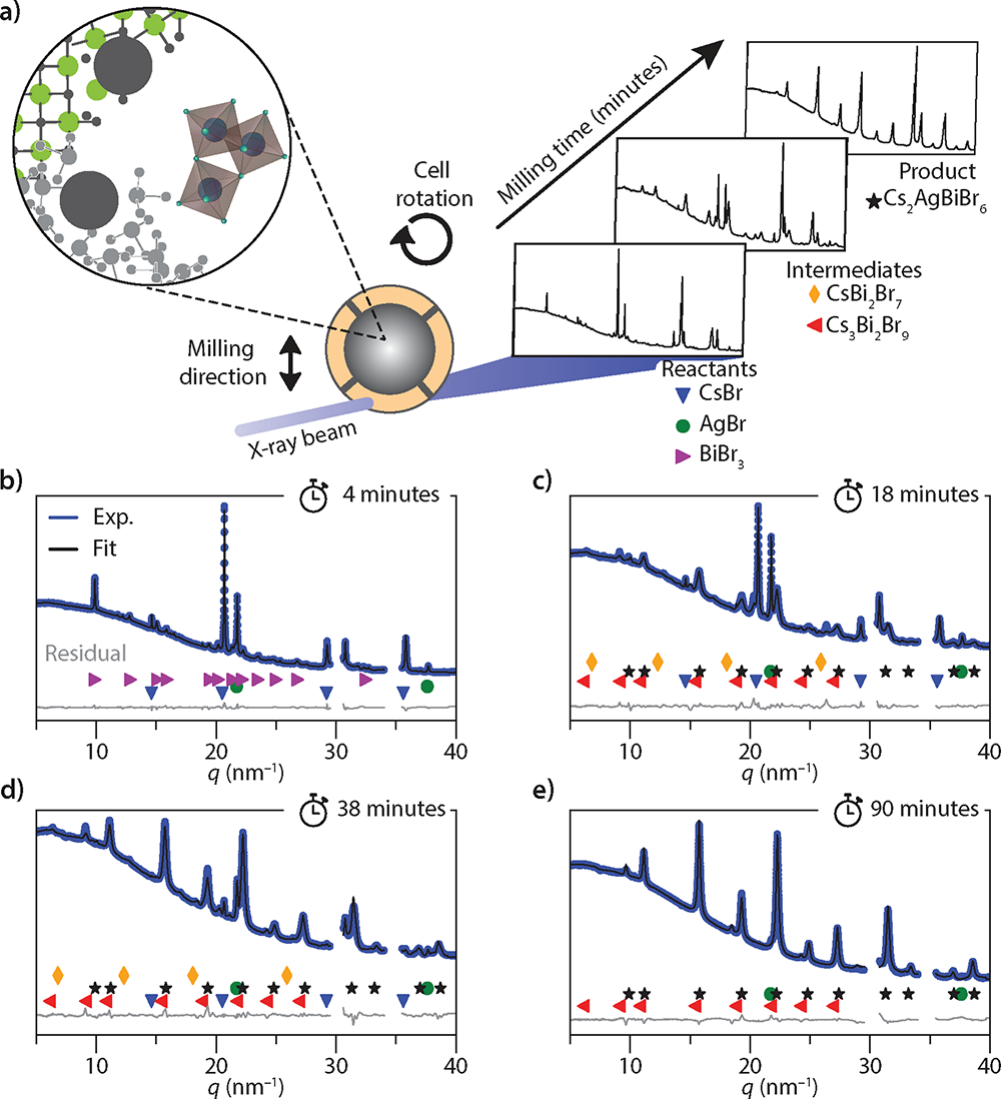
*In situ* ball milling and structure refinement. (a) Schematic of the *in situ* ball mill setup. The steel grinding beads and reactant powders are loaded in the inner compartment (in gray). An opening that is much smaller than the grinding beads diameter (7 mm) allows the reactants to move between the milling inner compartment and the probing outer compartment (in orange). For each reaction, the ball to powder ratio was 25:1. The cell is simultaneously shaken vertically (at 40 Hz) and rotated (at 5 Hz) so that the outer compartment is continuously refreshed. The walls of the outer compartment are transparent to X-rays, so that powder diffraction patterns can be collected over time. (b–e) XRD patterns as a function of *q*, for experimental data and refinement fits taken after (b) 4 minutes (only reactants), (c) 18 minutes (reactants, intermediates and product), (d) 38 minutes (reactants, intermediates and product), and (e) 90 minutes (product). The most intense reflections of each phase are indexed following the legend in (a). Note that $q=\frac{4\pi }{\lambda }\sin\left(\theta \right)$ with λ the X-ray wavelength and θ the diffraction angle.

Figure 1b–e shows three experimentally obtained XRD patterns (blue) and the corresponding refinement fits (black) for the mechanochemical synthesis of Cs_2_AgBiBr_6_ at different milling times. All diffraction patterns are plotted as a function of the scattering vector *q*, which is related to the diffraction angle, θ, by $q=\frac{4\pi }{\lambda }\sin\left(\theta \right)$ . To obtain information on the crystallization kinetics and identify crystalline intermediates, we refined all XRD patterns using the Rietveld method. Here, the fit parameters are the lattice constants, reflection width, a scaling factor, and a background (for further details see the Supporting Information).

Due to the large number of fitting parameters and experimental conditions, it is not straightforward to assess the quality of a fit based on common statistical measures such as the goodness-of-fit, χ^2^, or *R* factors.^42^ In this work, we therefore use the residual (experimental – fit) together with chemical and physical intuition to assess the outcome of the fits.

Upon starting the milling, the observed XRD pattern displays the reflections of the reactants CsBr, AgBr, and BiBr_3_ (Figure 1b). The patterns were refined using these three crystal structures as input, which provides a good match with the observed pattern, as reflected by the flat residual (gray). After a couple of minutes, see the 18 minutes pattern in Figure 1c, the reflections of three new crystal phases have appeared. These are attributed to CsBi_2_Br_7_ (127-phase) and Cs_3_Bi_2_Br_9_ (329-phase) as well as a small amount of the desired end product Cs_2_AgBiBr_6_ (2116-phase). The 329-phase is a stable intermediate that is typically observed as undesired side phase for various synthesis methods of Cs_2_AgBiBr_6_.^21,27,43^ The 127-phase, however, has not been reported before as an intermediate, and previous literature proposed Cs_3_BiBr_6_ (316-phase) as an intermediate phase upon *ex situ* characterization.^44^ We note that it is not straightforward to resolve the reflections of the 127- and 316-phase from the background (Figure S1). We therefore have a closer look at the reaction kinetics of the reactants to elucidate whether the Cs-rich 316-phase or the Bi-rich 127-phase is formed as the dominant reaction intermediate.

From refinement of the experimental XRD patterns, the molar fractions of the reactants, intermediates, and product are extracted (see Supplemental Note 2). Figure 2 shows the mole fractions of the intermediates CsBi_2_Br_7_ (orange), Cs_3_Bi_2_Br_9_ (red), and product Cs_2_AgBiBr_6_ (black) (Figure 2a), and the precursor salts (Figure 2b) as a function of milling time. After three minutes (first data point in Figure 2b), the reactants are homogenized resulting in a 2:1:1 CsBr:AgBr:BiBr_3_ molar ratio, consistent with the initial loading ratio into the grinding jar.

**Figure 2:**
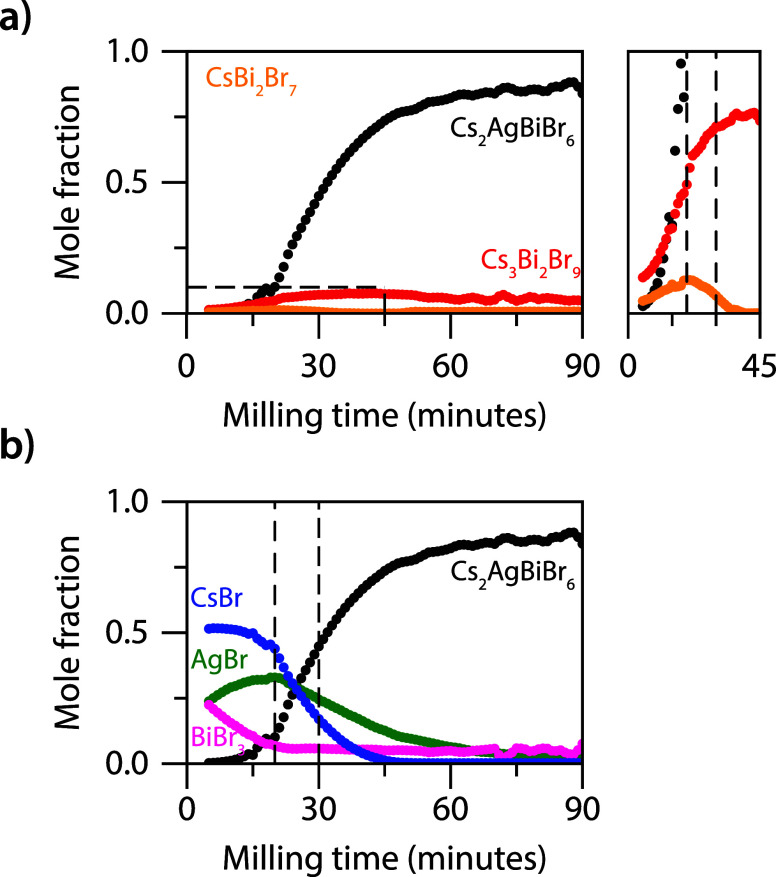
Formation kinetics of Cs_2_AgBiBr_6_. (a) Mole fractions of the 127- and 329-phase (orange and red, respectively) and Cs_2_AgBiBr_6_ (black) as a function of milling time. The zoom-in shows that the 127- and the 329-phase peak around 19 and 30 minutes, respectively. (b) Mole fractions of the reactants CsBr (blue), AgBr (green), and BiBr_3_ (magenta) and Cs_2_AgBiBr_6_ as a function of milling time. The immediate decrease in the BiBr_3_ concentration indicates the formation of a Bi-rich first intermediate. As the 127-phase peaks (dashed line at 20 minutes) the CsBr concentration decreases more rapidly indicating the formation of the 329- and elpasolite phase. The AgBr concentration starts to decrease as the 329-phase approaches its maximum presence around 30 minutes (dashed line).

In the first 15 minutes, the BiBr_3_ concentration drops significantly while the amount of AgBr and CsBr remains virtually constant (Figure 2a). The apparent increase in AgBr during this time window is likely due to the formation of amorphous compounds, resulting in a higher relative proportion of AgBr in the crystalline phases. As such, it is possible that the BiBr_3_ and CsBr concentrations are also initially somewhat overestimated. At the same time, on comparing the three precursor salts, it is evident that the biggest concentration drop occurs for BiBr_3_, followed by CsBr and last AgBr. To form the previously reported reaction intermediate 316-phase, a steep decrease of the CsBr concentration compared to BiBr_3_ would be expected, as

$$3\mathrm{CsBr}+{\mathrm{BiBr}}_{3}\to {\mathrm{Cs}}_{3}{\mathrm{BiBr}}_{6}$$ 1

The rapid drop in BiBr_3_ however (magenta curve in Figure 2b) supports the presence of the Bi-rich 127-phase intermediate rather than the Cs-rich 316-phase intermediate. The magnification in Figure 2a shows the evolution of the 127- (yellow) and 329- (red) phases. As the 127-phase peaks, around 19 minutes, the CsBr transformation curve starts to drop. This indicates the incorporation of CsBr into the 127-phase, forming the 329-intermediate. We therefore propose that the formation mechanism of Cs_2_AgBiBr_6_ proceeds *via:*

$$\mathrm{CsBr}+2{\mathrm{BiBr}}_{3}\to {\mathrm{CsBi}}_{2}{\mathrm{Br}}_{7}$$ 2a

$${\mathrm{CsBi}}_{2}{\mathrm{Br}}_{7}+2\mathrm{CsBr}\to {\mathrm{Cs}}_{3}{\mathrm{Bi}}_{2}{\mathrm{Br}}_{9}$$ 2b

$${\mathrm{Cs}}_{3}{\mathrm{Bi}}_{2}{\mathrm{Br}}_{9}+\mathrm{CsBr}+2\mathrm{AgBr}\to 2{\mathrm{Cs}}_{2}{\mathrm{AgBiBr}}_{6}$$ 2c

From our *in situ* XRD experiments, it follows that the trigonal 329-phase (*P*3̅*m*1) is the final intermediate before the cubic elpasolite phase is formed (Figure 2a). Close examination of the 3D representation of the triclinic 127-phase (*P*1̅, see Figure 3a) reveals that this unit cell comprises two [BiBr_3_]-octahedra that share one halide. Similar corner-sharing octahedra are found in the 329-scaffold (black ellipses in Figure 3a,b), so the 127-phase is a likely intermediate for the 329-phase. Although there may still be 316- (Figure S2) or other phases formed during the reaction, the observed reaction kinetics provide evidence for the 127-phase as a dominant first intermediate, that is subsequently transformed into the 329-phase.

**Figure 3:**
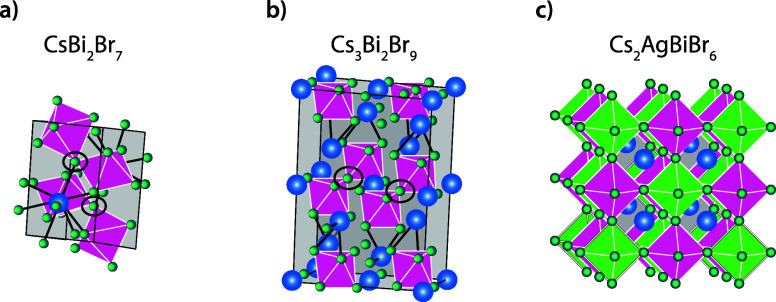
Crystal structures of intermediate phases in the mechanochemical synthesis of Cs_2_AgBiBr_6_. Schematic representation of the unit cells of (a) for the first intermediate CsBi_2_Br_7_, (b) for the second intermediate phase Cs_3_Bi_2_Br_9_, and (c) for the product Cs_2_AgBiBr_6_. For clarity the Cs–Br bonds are represented by black lines. The marked halides (with black ellipse) that are corner shared by the [BiBr_3_]-octahedra make CsBi_2_Br_7_ the most likely first intermediate in this reaction.

After 20 minutes, the 127-phase peaks (Figure 2a), which can be understood by the fact that the BiBr_3_ is fully depleted (Figure 2b), so that the formation of 127-phase (eq 2a) is no longer possible. Reaction of 127- into 329- results in disappearance of this 127-phase intermediate after 30 minutes. Hence, the 329-phase can no longer be formed at this point (eq 2b), and the presence of the 329-phase approaches its maximum. Only at this point, we observe that the AgBr concentration starts to drop, together with a further depletion of the CsBr precursor. This point marks the start of the final reaction step (eq 2c) and the formation of the elpasolite structure (*Fm*3̅*m*, Figure 3c). The potential presence of amorphous phases complicates a quantitative description of the entire reaction mechanism. However, as this reaction concerns solids that are ground in a closed system, we can assume that no material is lost during the reactions. Therefore, we will use the transformation curve of Cs_2_AgBiBr_6_ to gain insight into the type of crystal growth and the formation kinetics during a mechanochemical reaction, as discussed below.

Over the entire experiment, the formation curve of Cs_2_AgBiBr_6_ has a sigmoidal shape, which is typically observed for nucleation and growth transformation kinetics.^45^ As such, the formation kinetics can be described according to Kolmogorov–Johnson–Mehl–Avrami theory,^46^ known as the Avrami equation:

$$f\left(t\right)=1-\exp\left(-kt^{n}\right)$$ 3

with *f* the weight fraction of the final product at milling time *t*, *k* the conversion rate constant, and *n* the Avrami index. Here, *n* typically ranges between 1 and 4 and comprises information on the type of crystal growth.^45,47–49^ The value of *n* is typically determined by plotting log­(*t*) as a function of log­(log­(1/1–*f*)) (with *f* the weight fraction of the product), and the slope of the resulting curve equals *n* (Figure S3a,b). We, however, note that Avrami theory assumes a constant nucleation rate over the entire reaction medium, which may not be the case in mechanochemistry. Thus, care must be taken with the interpretation of *n*. For Cs_2_AgBiBr_6_ we find that *n* ≈ 3.4 suggesting diffusion-controlled growth at a conversion rate *k* = 0.21 × 10^–4^ min^–1^. A similar *n*-value has been observed for typical solvent-based (re)­crystallization of Cs_2_AgBiBr_6_ microcrystalline powders or single crystals.^50^ Therefore, the growth and nucleation rates of this mechanochemical reaction are similar to those of solvent-based routes, providing a solvent-free and equally fast alternative for the formation of microcrystalline Cs_2_AgBiBr_6_.

After ca. 40 minutes of milling, during the incorporation of AgBr into the 329-phase to form the final product, we observe a change of the diffraction peak shapes corresponding to the elpasolite phase. The diffraction peak width is affected by the crystalline domain size as well as the presence of microstrain, i.e., local variations in *d*-spacing due to compressive or tensile strain.^51^ We used Williamson–Hall (WH) plots (Figures S4–S8) to derive both the microstrain ε and crystalline domain size *L* over time. Note that the peak widths have first been corrected for instrumental broadening, as detailed in Supplemental Note 3. From the WH plots, we find that the Cs_2_AgBiBr_6_ exhibits isotropic microstrain of ε ∼ 0.005 after ca. 40 minutes of milling, which is in line with the suggested diffusion-controlled growth mechanism (see Supplemental Note 3). It seems likely that this microstrain originates from local expansions and contractions of the lattice as ions diffuse toward their final position. Consequently, the strain gradually relaxes over time and stabilizes at ε ∼ 0.0025 after approximately 60 minutes, when the reaction is close to completion. The residual microstrain could be related to crystallographic defects, resulting in local lattice distortion. Furthermore, the WH plots suggest that the crystalline domain size slightly increases to ca. 30 nm after 100 minutes of milling. Larger crystalline domain sizes could be achieved by thermal annealing.

To study the mechanochemical formation kinetics of mixed-cation Cs_2_AgBi_0.5_M_0.5_Br_6_ elpasolites, we replaced half of the BiBr_3_ precursor with SbBr_3_, InBr_3_, or FeBr_3_. The XRD patterns collected at the end of the synthesis confirm the formation of the elpasolite phase (Figure 4a). The zoom-in reveals a shift of the XRD patterns to larger values of *q*, indicating a reduction of the unit cell volume, due to the smaller ionic radii of Sb^3+^ (0.76 Å), In^3+^ (0.80 Å), and Fe^3+^ (0.65 Å) compared to Bi^3+^ (1.03 Å).^52^ Consistent with earlier reports, alloying yields materials with bandgaps of 1.8 eV (Sb^3+^),^26^ 2.3 eV (In^3+^),^26^ and 1.1 eV (Fe^3+^)^11,35,53^ (Figure 4b). These bandgap changes are reflected by changes in the powder colors; see photograph displayed in Figure 4c.

**Figure 4:**
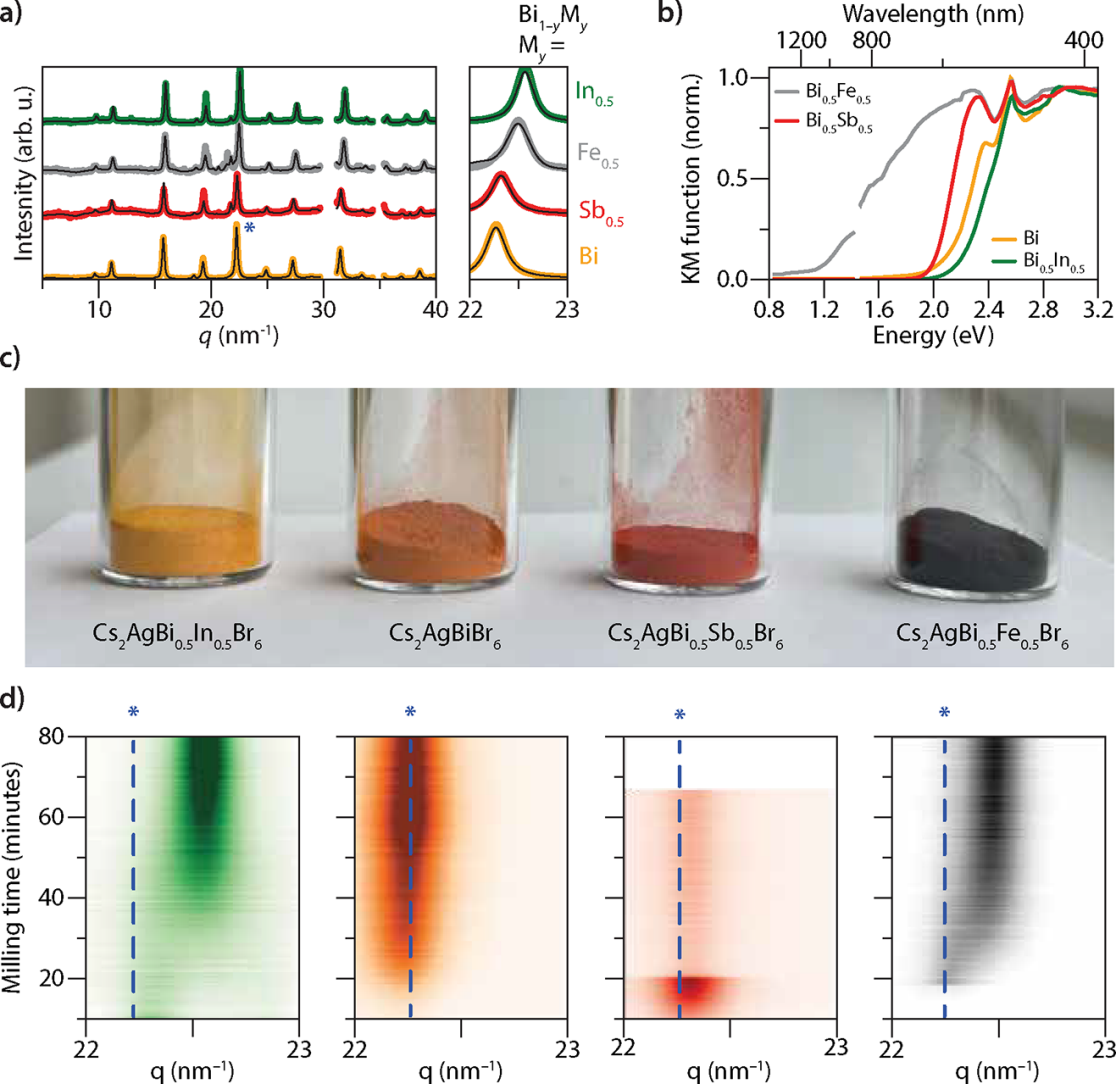
Alloyed Cs_2_AgBi_0.5_M_0.5_Br_6_compositions. (a) XRD patterns of alloyed Cs_2_AgBi_0.5_M_0.5_Br_6_ compositions with M = In, Bi, Sb, and Fe confirming the elpasolite crystal structure. The zoomed-in view shows the shift of the 400-reflection as a result of the smaller unit cell upon alloying. (b) The Kubelka–Munk transform provides an approximation of the absorption profile of the alloyed elpasolite compositions as a function of photon energy and wavelength. (c) The significant change in visible light absorption upon alloying is highlighted by the photographs. (d) Heat maps of the 400-reflection of the different elpasolite alloys as a function of the milling time. For M = In^3+^ and Fe^3+^, a shift of the reflection is observed between 10 and 40 minutes, indicating a reduction of the unit cell volume. The blue dashed line is a guide to the eye centered at the maximum of the 400-reflection of Cs_2_AgBiBr_6_.

For alloying with Sb^3+^ and In^3+^, no major side-phases are present in the XRD patterns, indicating complete conversion. Notably, in spite of the similar ionic radii of In^3+^ and Sb^3+^, the lattice parameter of −Bi_0.5_In_0.5_– (11.141 Å) is smaller than that of −Bi_0.5_Sb_0.5_– (11.251 Å). This smaller lattice parameter may be due to the more ionic character of the In–Br bond compared to Sb–Br. For partial replacement of Bi^3+^ with Fe^3+^, we estimate that the lattice parameter after 90 minutes of milling corresponds to 43% of Fe^3+^ by extrapolating the previously reported size curve^11^ (Figure S9). In line with this, some FeBr_3_ is still observed in the reaction mixture. The incorporation threshold of ∼ 43% Fe^3+^ can be understood from geometric constraints, as Fe^3+^ is much smaller than Bi^3+^. Interestingly, when alloying with In^3+^ and Fe^3+^, the reflections characteristic for the elpasolite crystal structure shift as a function of milling time (Figure 4d and Figure S10). For these compositions, the elpasolite reflections gradually shift to larger diffraction angles in the first 40 minutes. On the other hand, the elpasolite reflections for the Sb^3+^ alloy and full Bi composition remain at a constant *q*-value. These observations show that the type of M substituent affects the formation pathway of alloyed AgBi-elpasolites.

To study the formation kinetics, we perform a similar refinement analysis as described above (Figures S11–S13). Partial replacement of BiBr_3_ with MBr_3_ (with M = Sb^3+^, In^3+^, or Fe^3+^) to the grinding jar leads to additional crystalline phases. Due to the large variety of potential intermediates and side phases, all with unknown alloying ratios (and thus peak positions), we cannot unravel the nature of the reaction intermediates. We therefore only consider the final elpasolite product. Figure 5a shows the weight fraction of the pristine (Bi) and alloyed (Sb_0.5_, Fe_0.5_, and In_0.5_) elpasolites as a function of milling time. Upon alloying with Sb^3+^, the transformation curve has a sigmoidal shape, which can be described using eq 3 with *n*_*Sb*_ = 2.8 (Figures S3c,d) and a conversion rate of *k*_Sb_ = 6.1 × 10^–4^ min^–1^.

**Figure 5:**
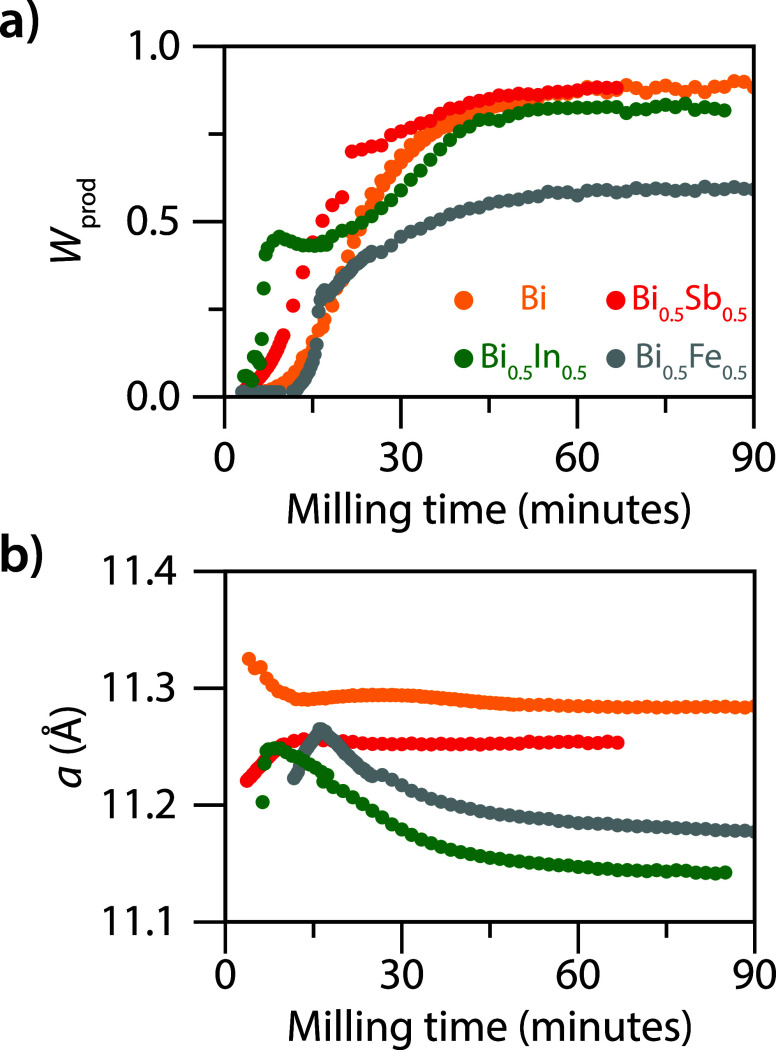
Formation mechanism of alloyed Cs_2_AgBi_1–*y*_M_*y*_Br_6_. (a) Weight fraction of the elpasolites alloys as a function of milling time. (b) Lattice parameter, *a*, of the elpasolite phase as a function of milling time.

As discussed above, for Sb^3+^ alloying, the lattice parameter of the elpasolite phase is reduced with respect to the Bi-based counterpart (Figure 5b). While the relative amount of Cs_2_AgBi_0.5_Sb_0.5_Br_6_ phase increases until ∼60 minutes, we find that the lattice parameter of this alloyed elpasolite phase remains constant during the entire reaction (Figure 5b). These observations imply that the 329-phase is already alloyed in the final Sb:Bi ratio (Figure S14), reacting with AgBr and CsBr to form into the elpasolite product. Such alloyed Sb-based 329-phases have been reported in the literature as well.^33^

Notably, a different formation mechanism is observed when alloying with Fe^3+^ or In^3+^. In contrast to alloying with Sb^3+^, the lattice parameters of Cs_2_AgBi_1–*y*_Fe_*y*_Br_6_ (final *y* = 0.43) and Cs_2_AgBi_1–*y*_In_*y*_Br_6_ (final *y* = 0.5) gradually decreases over time (Figure 5b). Since this decrease was not observed for the other compositions, the lattice contraction during ball milling is likely due to an increase in *y* during the reaction. Therefore, we propose that smaller Fe^3+^ or In^3+^ substituents are incorporated in an additional reaction step. The incorporation of InBr_3_ as a final step is further supported by the decreasing intensity of the most intense reflections of the InBr_3_ phase at 10.0 nm^–1^ (001-plane), 22.9 nm^–1^ (131-plane), and 32.69 nm^–1^ (202-plane) (Figure S15). Between 10 and 40 minutes, the lattice parameter of the elpasolite phase decreases, i.e., the elpasolite reflections shift to larger *q*-values. At the same time, the intensity of the InBr_3_ reflections drop considerably (Figure S15). After 40 minutes, the InBr_3_ reflections cannot be distinguished from the background, which coincides with the time scale at which the lattice parameter of the elpasolite phase remains constant (Figure 5b). Moreover, the contribution of the elpasolite phase to the diffraction pattern remains constant as well (Figure 5a). These observations indicate that the depletion of the InBr_3_ phase is accompanied by a reduction of the lattice constant of the elpasolite phase.

Similar to alloying with In^3+^, the formation of Cs_2_AgBi_1–*y*_Fe_*y*_Br_6_ comes with a gradual reduction of lattice parameter *a*. Despite the smaller ionic radius of Fe^3+^ compared to In^3+^, the lattice parameter of Cs_2_AgBi_1–*y*_Fe_*y*_Br_6_ remains larger than that for Cs_2_AgBi_1–*y*_In_*y*_Br_6_. We again note that the conversion of Cs_2_AgBi_0.5_Fe_0.5_Br_6_ is incomplete and that, based on our refinements, only about 50 wt % of the probed analyte has the elpasolite crystal structure after 90 minutes of ball milling. As such, a significant contribution of the 329-phase is observed in the diffraction pattern (Figure 4a and Figure S13). These observations suggest the incomplete incorporation of Fe^3+^, which is in line with our previous work.^11^ Nevertheless, the incorporation of Fe^3+^ and In^3+^ clearly follows a different reaction mechanism compared with the Bi- and Sb-based compositions. Moreover, WH-plots of the pristine and alloyed elpasolites show similar crystalline domain sizes and strain evolution during the reaction. The residual strain suggests defect-rich compositions, without a notable difference between the pristine and alloyed compositions (see Supplemental Note 3).

The Avrami indices of the additional step for both In^3+^- and Fe^3+^-alloying are *n*_In_ = *n*_Fe_ = 2.5, with conversion rates of *k*_In_ = 1.5 × 10^–4^ min^–1^ and *k*_Fe_ = 2.0 × 10^–4^ min^–1^ (Figure S3e–h), approaching *n* values that are associated with preferential growth mechanisms.^45,47,54,55^ We note that the relative ionic radius is most likely not the rate determining factor since the trend in ionic radii of Bi^3+^ > In^3+^ ∼ Sb^3+^ > Fe^3+^ is not in line with the observed trend for the formation mechanisms.^52^ Moreover, in previous work, we found that the lattice flexibility is hardly affected by the type of trivalent metal, and therefore also the mechanical properties probably play a minor role in the reaction mechanism.^12^

To rationalize the difference in formation mechanisms, we instead consider the intermediate phases. In the final reaction step to form Cs_2_AgBiBr_6_ and alloyed Bi_0.5_Sb_0.5_, the AgBr is incorporated into the 329-phase, which is known to be stable across the full compositional range between Cs_3_Bi_2_Br_9_ and Cs_3_Sb_2_Br_9_.^33^ As such, the 329-phase may already be alloyed in the right stoichiometry for forming the Bi_0.5_Sb_0.5_ elpasolite. For In^3+^ and Fe^3+^, however, only the chloride analogues of the 329-phases have been reported,^56,57^ and we could not find any reference to bromide 329-phases. This implies the absence of alloyed 329-phases as intermediates for Bi_0.5_In_0.5_ and Bi_0.5_Fe_0.5_ elpasolites. We note that Bi and Sb have similar electronegativities (χ_Bi_ = 2.02 and χ_Sb_ = 2.05), and also In and Fe have similar but lower χ-values (χ_In_ = 1.78 and χ_Fe_ = 1.83).^58^ We, therefore, hypothesize that In^3+^ or Fe^3+^ is incorporated into the elpasolite phase (Cs_2_AgBiBr_6_) via a cation exchange step. This might also explain why, e.g., Fe-alloyed elpasolite thin films have not yet been synthesized by using solvent-based methods. In solvent-based (nano)­crystal synthesis, separating the crystal formation from the alloying or doping step is a well-established approach for obtaining metastable phases.^22,59–62^ The mechanochemical synthesis method presented here provides a route to directly access these alloyed compositions.

## Conclusions

In this work, we studied the mechanochemical formation mechanism of alloyed AgBi-elpasolites. *In situ* ball milling experiments and Rietveld refinements fits, considering multiple phases including reactants, intermediates, and product, revealed the mechanochemical reaction mechanism and kinetics of Cs_2_AgBiBr_6_. Our *in situ* XRD studies reveal that the first step in Cs_2_AgBiBr_6_ formation proceeds via a Bi-rich intermediate (CsBi_2_Br_7_, 127-phase). This is underlined by an initial fast decrease in the BiBr_3_ contribution to the XRD pattern. Subsequently, the presence of CsBr decreases when the second intermediate, Cs_3_Bi_2_Br_9_ or 329-phase, is formed. In the final reaction step, AgBr is incorporated into the 329-phase to obtain Cs_2_AgBiBr_6_. Next, we studied the formation mechanism of alloyed Cs_2_AgBi_1–*y*_M_*y*_Br_6_ with M = Sb^3+^, In^3+^, and Fe^3+^. For alloying with Sb^3+^, a similar reaction pathway is observed in which an Sb^3+^-alloyed intermediate and subsequently an elpasolite phase are immediately formed. In contrast, when alloying with Fe^3+^ and In^3+^ an additional final alloying step is observed. Analysis of the lattice space parameters of the elpasolite phases shows that first pristine Cs_2_AgBiBr_6_ is formed, after which Fe^3+^ and In^3+^ are incorporated in a fourth step. The incorporation of smaller Fe^3+^ or In^3+^ cations gradually reduces the lattice parameter of the elpasolite phase over time. We hypothesize that these elpasolites do not immediately form as alloys due to the absence of alloyed 329-phase as reaction intermediate. Overall, the mechanochemical formation of (alloyed) Cs_2_AgBiBr_6_ proceed on similar time scales as typical solvent-based synthesis routes. This means that ball milling provides a solvent-free route to form microcrystalline elpasolite solid solutions. Hence, these insights provide pathways toward designing new, complex materials with highly tunable properties.

## Supplementary Material



## Notes

Experimental methods, additional data, and fitting procedures (PDF)

## 2

Current affiliation: École Polytechnique Fédéral de Lausanne, Rue de l’industrie 17, 1951, Sion, Switzerland

## 3

Current affiliation: Vrije Universiteit Amsterdam, De Boelelaan 1105, 1081 HV Amsterdam, The Netherlands
