# Supplementary material for: Mechanochemical Formation Mechanism of Alloyed AgBi-Elpasolites
Source: J Am Chem Soc. 2025 Jul 3;147(28):24519–26. doi: 10.1021/jacs.5c05045 (PMC12272686; doi:10.1021/jacs.5c05045)
Supplement: Supplementary file 1 [file ja5c05045_si_001.pdf]

Supporting Information

Mechanochemical formation mechanism of alloyed AgBi-elpasolites

Huygen J. Jöbsis[1,2+], Loreta A. Muscarella[1,3+], Michał Andrzejewski[4], Nicola P.M. Casati[4], and Eline M. Hutter*[1]

1) Department of Chemistry, Utrecht University, Princetonlaan 8, 3584 CB Utrecht, The Netherlands
2) Current affiliation: École Polytechnique Fédéral de Lausanne, Rue de l'industrie 17, 1951 Sion, Switzerland
3) Current affiliation: Vrije Universiteit Amsterdam, De Boelelaan 1105, 1081 HV Amsterdam, The Netherlands
4) Swiss Light Source, Paul Scherrer Institute, Forschungstrasse 111, 5232 Villigen, Switzerland

+Authors contributed equally

• Current address

*Corresponding author: e.m.hutter@uu.nl

Supplemental note 1 – Experimental Methods

Chemicals. The elpasolite powders are prepared using cesium bromide (CsBr; TCI, purity > 99%), silver (I) bromide (AgBr; Alfa Aesar, Premion®, purity 99.998% metals basis), bismuth (III) bromide ($BiBr_3$; Merck, purity ≥ 98%), indium (III) bromide ($InBr_3$, Alfa Aesar, purity 99.99% (metal basis)), and antimony (III) bromide ($SbBr_3$, Alfa Aesar, purity ultra-dry 99.999% (metal basis)), iron (III) bromide ($FeBr_3$, Alfa Aesar, purity >98%). The precursor powders are obtained weighing the components with the desired molar stoichiometric ratio. The BiX_3 precursor salt were heated at 150 °C for at least one hour.

Diffuse reflectance UV–vis spectroscopy. Diffuse reflectance spectra were obtained using a Perkin–Elmer lambda UV–Vis–NIR–lambda950S spectrometer equipped with an integrating sphere. The background was obtained by placing the sample holder filled with PTFE (400 µm grain size) at the sample position. The spectra were recorded from 1500 to 350 nm with a step size of 2 nm and an integration time of 0.4 s. The data around 1.42–1.46 eV (850–870 nm) is left out due to an artifact of the experimental setup.

***In situ* X-ray diffraction during ball mill synthesis.** A stochiometric mixture of the precursor salts — *i.e.* CsBr, AgBr, $1–y$ $BiBr_3$ and y $M$$Br_3$ (with M = Sb, In, and Fe) — was loaded in the inner compartment of the grinding jar together with two steel balls (7 mm in diameter). For each reaction the total mass of the reactants was *ca.* 1 g resulting in a ball to powder ratio (BPR) of 25:1. The grinding jar was simultaneously shaken veritcally (at 40 Hz) and rotated (at 5 Hz), so that the outer probing compartment was refreshed continuously. Every 20 s an XRD pattern was recorded.

The *in situ* XRD data during a ball mill synthesis were collected at the X04SA Materials Science (MS) beamline at the Swiss Light Source, Paul Scherrer Institute (PSI, Villigen).[1] The wavelength (λ = 0.49178 Å) was calibrated with a NIST standard (SRM 660b, LaB6) using the high resolution 1D diffractometer once the beam is ready. With the use of an X-ray camera the beam was aligned with respect to the probing compartment so that it hit the probing compartment most of the time during milling. The beam is focused and slitted down to some 150×150 microns. The position of the detector (Pilatus 6M) is calibrated using Dioptas software.[2] Once the equipment is ready a standard in the same configuration as the sample is run to obtain the instrumental resolution function and the detector position.

Supplemental note 2 – Structure refinement

Rietveld Refinement method. The refinements using the Rietveld method was performed using the FullProfSuite software. All diffractograms were fitted using Pseudo–Voigt functions with the unit cell dimensions, reflection intensity as variables. In some cases a shape factor was used as variables to improve the quality of the fits. Note that including these variables only lower the residuals, without changing the lattice parameters obtained from the fits. The CIFs that were used for the Rietveld refinements were extracted from the ICSD database (**Table 1**).

Weight- and molar fraction calculations. To determine the contribution of each crystalline phase to the XRD pattern a scaling factor, S_j, is included in the refinement iterations. With S_j the weight fraction, f_j, of each phase j is determined using:

$$f_j = \frac{\frac{S_j * Z_j * M_j * V_j}{t_j}}{\sum_i S_i * Z_i * M_i * V_i}$$

with Z_j the number of ions per unit cell, M_j the mass of the ions in the unit cell, V_j the unit cell volume and t_j an X-ray absorption contrast factor (the Brindley factor).[3] W_j represents the relative mass of each crystalline phase to the total mass of the probed region.

We thus note that amorphous materials, which are likely present during a chemical reaction, are unaccounted for and will be discussed in more detail below. To study the reaction kinetics, we convert f_j to molar fractions (**Fig. 4.2**).

Composition	ICSD entry
$Cs_2AgBiBr_6$	252164
$Cs_2AgBi_{0.5}In_{0.5}Br_6$	252164*
$Cs_2AgBi_{0.5}Sb_{0.5}Br_6$	252164*
$Cs_2AgBi_{0.5}Fe_{0.5}Br_6$	257164*
$CsBi_2Br_7$	430595
Cs_3BiBr_6	12760
$Cs_3Bi_2Br_9$	1142
CsBr	22174
AgBr	53850
$BiBr_3$	100294

Table S1 — Compositions and corresponding ICSD entries used for refinement fits.

Comparison 316-, 127-, 329-, elpasolite-phase.

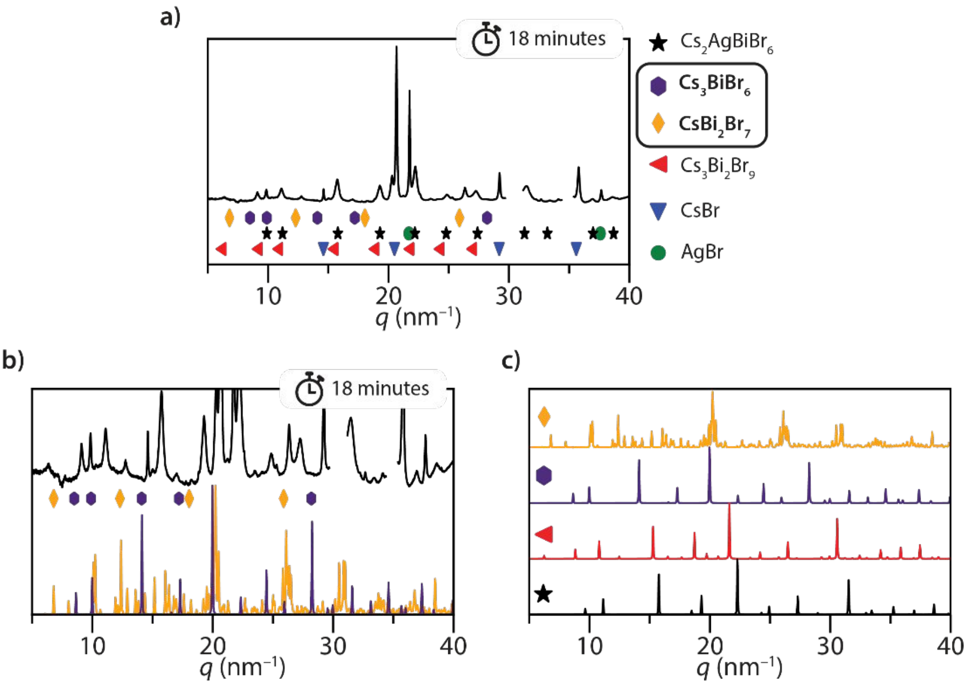

Figure S1 — Comparison 127- and 316-phase. a) Experimental XRD pattern after 18 minutes of ball milling. The Bragg positions of the most intense reflection of the reactants, intermediates and product are indexed. For clarity a manually selected background is subtracted. **b)** Zoom in of the experimental data presented in a) plotted together with the theoretical diffraction patterns of the 127- (orange) and 316-phase (indigo). **c)** Calculated diffraction patterns of the 127-, 316-, 329-phase (red), and $Cs_2AgBiBr_6$ (black).

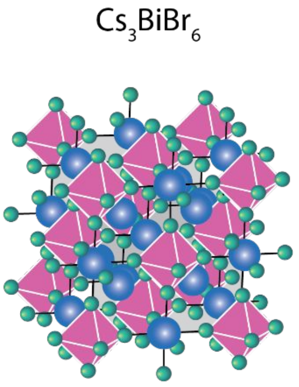

Figure S2 — Schematic representation of the 316-phase crystal structure. For clarity the Cs–Br bonds are represented by black lines. The octahedra around the Bi^{3+} cations are indicated in pink.

Fitting Avrami transformation curve. The Avrmi index, n, was determined by extracting the slope when plotting log(log(1/(1−f))) as a function of log(t). By plugging the extracted n-value in **Eq. 1** the conversion rate constant was determined. We again note that the care must be taken when interpreting this rate constant as our analysis only includes the transformation of crystalline phases. The presence of amorphous material might influence the observed reaction kinetics.

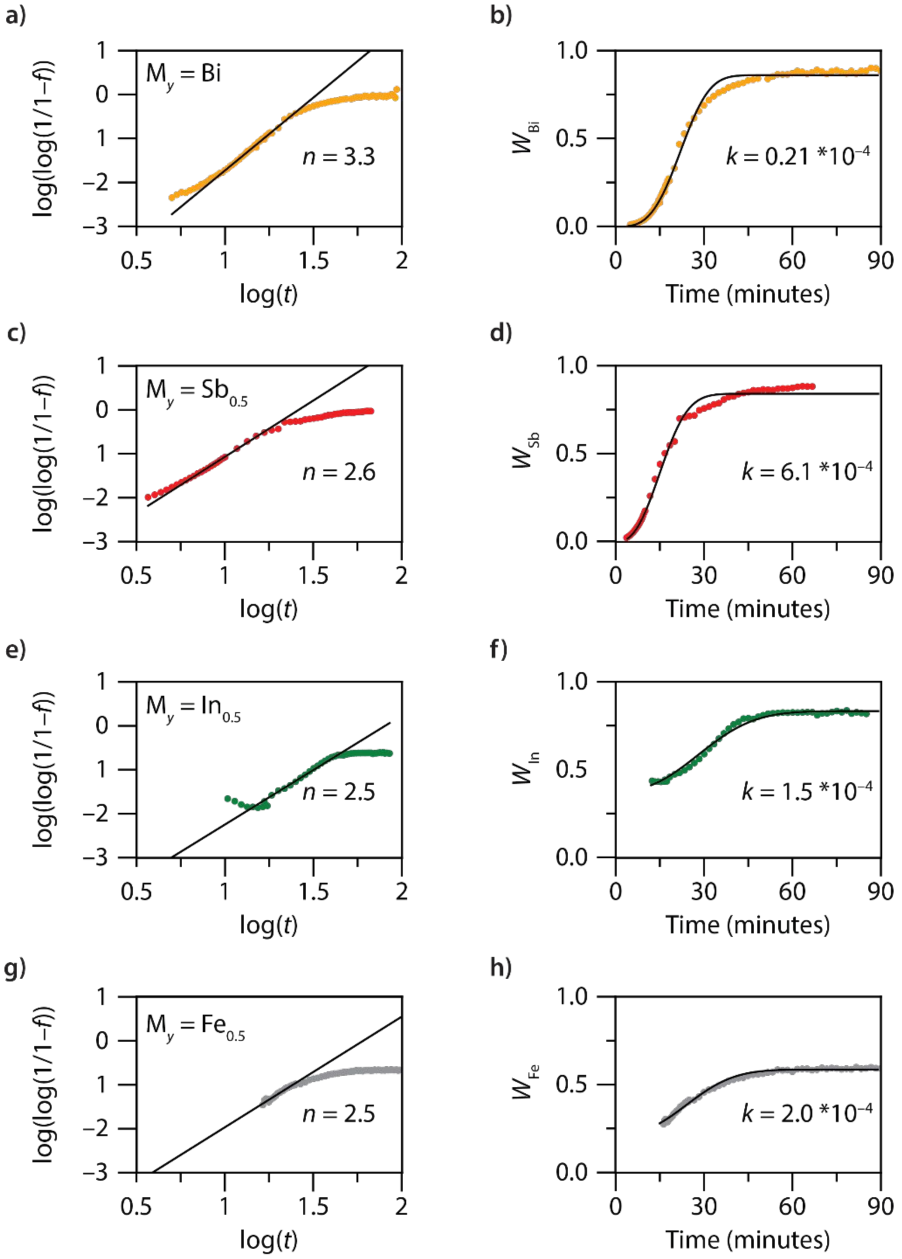

Figure S3 — Avrami plots of alloyed $Cs_2AgBi_{1-y}M_yBr_6$. Avrami plots to extract n for M_y = **a)** Bi, **c)** $Sb_{0.5}$, **e)** $In_{0.5}$, and **g)** $Fe_{0.5}$. By plugging the extracted n-values in **Eq. 4** the rate constants, k, are determined for M_y = **b)** Bi, **d)** $Sb_{0.5}$, **f)** $In_{0.5}$, and **h)** Fe0.5.

Supplemental note 3 — Strain and crystalline domain

From the line shape of the Bragg reflections information on the crystalline domain size and strain of the formed elpasolite materials can be extracted.[4] To do so, it is important to determine the instrumental broadening in particular because of the moving sampling volume (shaking probe compartment). This was done by recording the diffraction pattern of LaB_6 in a moving grinding jar, shaking at the same experimental frequency (40 Hz). Lorentzian functions are fit to determine the FWHM and peak positions of the LaB_6 reflections (**Fig. S1a**). To model the instrumental broadening over the entire probing range we fit a Caglioti function:

$$\beta^2_{instrumental} = U * tan^2(\theta) + V * tan(\theta) + W \qquad \text{(Eq. S1)}$$

with β the FWHM (in radians), θ the diffraction angle (in radians), and fitting parameters U, V, W (**Fig. S4b**).

Next, we fit pseudo-Voigt functions to different elpasolite reflections (**Figs. S2a–5a**) and correct the FWHM for instrumental broadening following:

$$\beta_{corrected} = \sqrt{\beta^2_{measured} - \beta^2_{instrumental}} \qquad \text{(Eq. S2)}$$

We note that for all elpasolite compositions, the peak shapes are best described for a pseudo-Voigt mixing parameter η = 1, *i.e.,* gaussian line profiles. In the literature, this is typically observed for strained materials, whereas less strained materials, such as LaB_6, are better described using Lorentzian line profiles (η = 0).[5]

With the FWHMs corrected for instrumental broadening, we derived the strain and crystalline domain size using Williamson-Hall (WH) plots (**Figs. S6b–9b**).[6] According to WH the strain (ϵ) and crystalline domain size (L) are related following:

$$\beta * \cos\theta = \frac{K\lambda}{L} + 4\epsilon * \sin\theta \qquad \text{(Eq. S3)}$$

with K a shape factor, λ the X-ray wavelength (0.49178 Å) and θ the reflection angle (in radians). As such, with the use of a linear fit and assuming K = 0.9 we extracted ϵ and L as a function of milling time (**Figs. S2b–d to Figs. 5b–d**).

Upon formation of the elpasolite phase, we observe a narrowing of the line shapes over time indicating an increase of the crystalline domain size. At the same, the strain is decreased and plateaus after *ca.* 60 minutes.

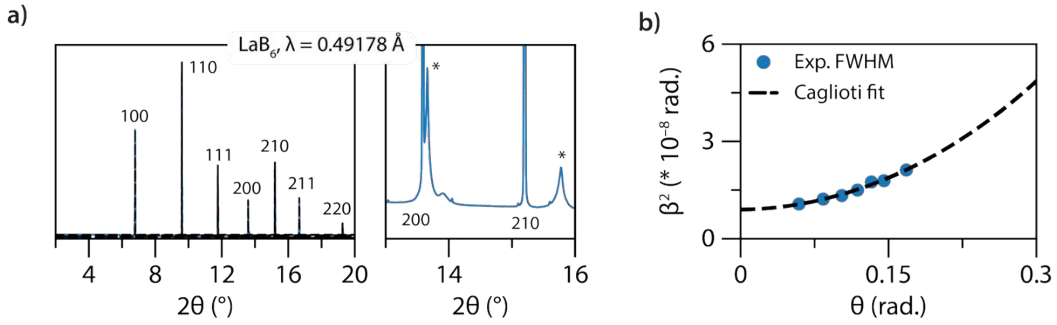

Figure S4 — Instrumental broadening correction. a) X-ray diffraction pattern of LaB_6 recorded in a shaking grinding jar. The *100-*, *110-*, *111-*, *200-*, *210-*, *211-*, *220-*reflections are fit using Lorentzian functions to determine the peak positions and FWHM of each reflection. The zoom in shows the reflections by the grinding jars indicated with an asterisk. **b)** The determined FWHM are shown as a function of reflection position and fit with a Caglioti function with $U = 1.594 * 10^{-6}$, $V = 1.941 * 10^{-8}$, $W = 3.576 * 10^{-8}$.

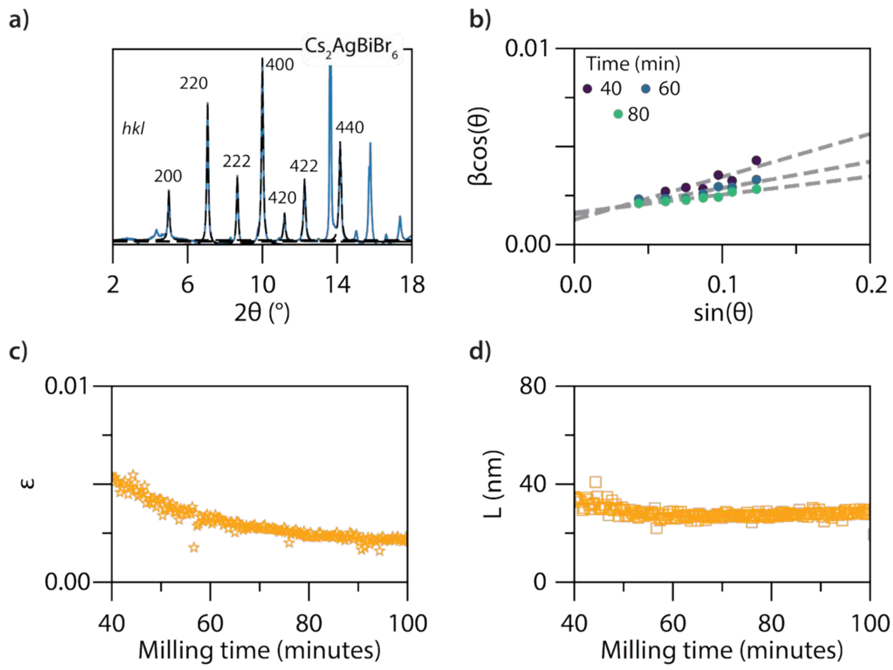

Figure S5 — Strain and crystalline domain in $Cs_2AgBiBr_6$ powders. a) Pseudo-Voigt functions are fit to seven Bragg-reflections of $Cs_2AgBiBr_6$ corresponding to the *200*-, *220*-, *222*- - *400*-, *420*-, *422*-, *440*-planes. Note that the wavelength of the X-ray radiation is 0.49178 Å. **b)** The WH plots present the FWHM as a function of diffraction angle is plotted for different milling times. **c)** The slope of a linear fit to the WH plot is used to determine the strain and **d)** the crystalline domain size.

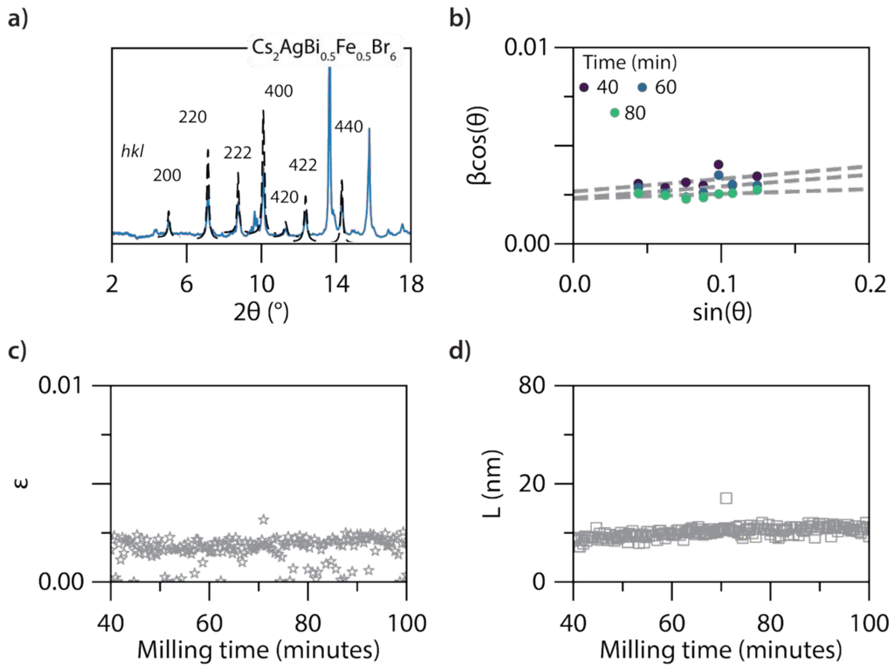

Figure S6 — Strain and crystalline domain in $Cs_2AgBi_{0.5}Fe_{0.5}Br_6$ powders. a) Pseudo-Voigt functions are fit to seven Bragg-reflections of $Cs_2AgBi_{0.5}Fe_{0.5}Br_6$ corresponding to the *200*-, *220*-, *222*-, *400*-, *420*-, *422*-, *440*-planes. **b)** The WH plots present the FWHM as a function of diffraction angle is plotted for different milling times. **c)** The slope of a linear fit to the WH plot is used to determine the strain and **d)** the crystalline domain size.

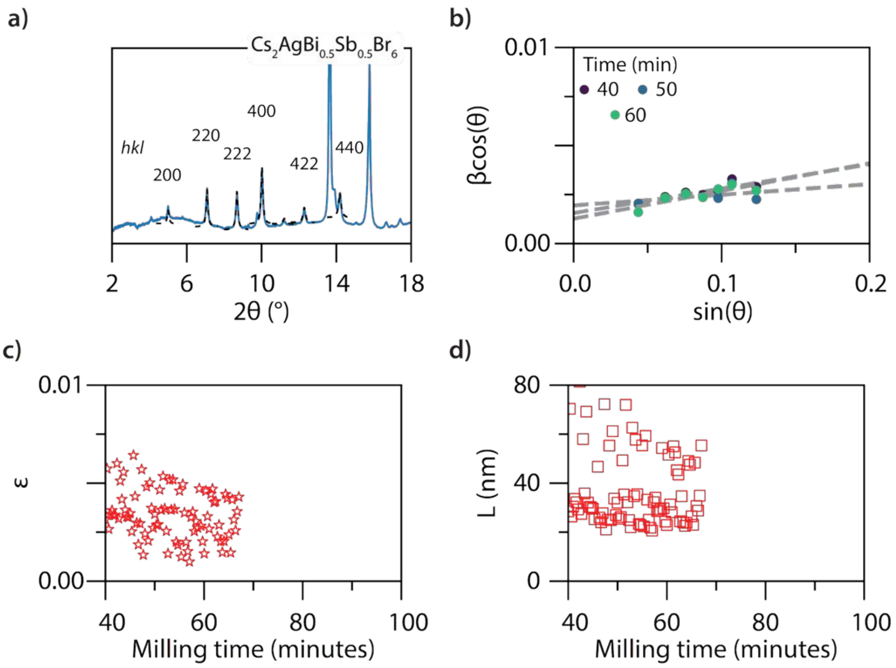

Figure S7 — Strain and crystalline domain in $Cs_2AgBi_{0.5}Sb_{0.5}Br_6$ powders. a) Pseudo-Voigt functions are fit to seven Bragg-reflections of $Cs_2AgBi_{0.5}Sb_{0.5}Br_6$ corresponding to the *200-*, *220-*, *222-*, *400-*, *420-*, *422-*, *440*-planes. **b)** The WH plots present the FWHM as a function of diffraction angle is plotted for different milling times. **c)** The slope of a linear fit to the WH plot is used to determine the strain and **d)** the crystalline domain size.

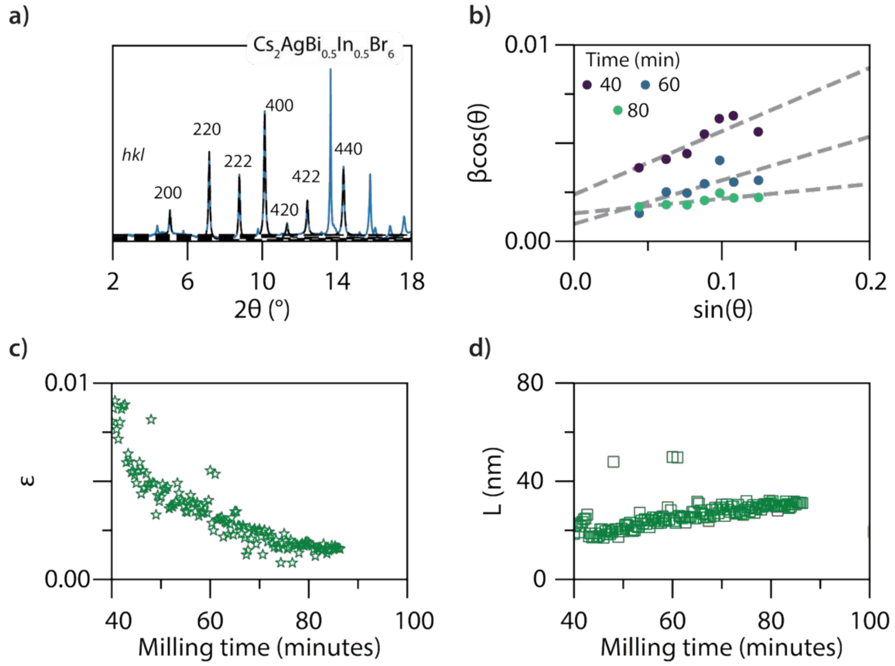

Figure S8 — Strain and crystalline domain in $Cs_2AgBi_{0.5}In_{0.5}Br_6$ powders. a) Pseudo-Voigt functions are fit to seven Bragg-reflections of $Cs_2AgBi_{0.5}In_{0.5}Br_6$ corresponding to the *200-*, *220-*, *222-*, *400-*, *420-*, *422-*, *440*-planes. **b)** The WH plots present the FWHM as a function of diffraction angle is plotted for different milling times. **c)** The slope of a linear fit to the WH plot is used to determine the strain and **d)** the crystalline domain size.

Figure S9 — Extrapolated Fe-content $Cs_2AgBi_{0.5}Fe_{0.5}Br_6$. The lattice parameters determined for $Cs_2AgBi_yFe_{1-y}Br_6$ reproduced from ref[7] are plotted as a function of the Fe-content. The dotted line is a linear function fit that was used to extrapolate the Fe-content when stoichiometrically milling $Cs_2AgBi_{0.5}Fe_{0.5}Br_6$.

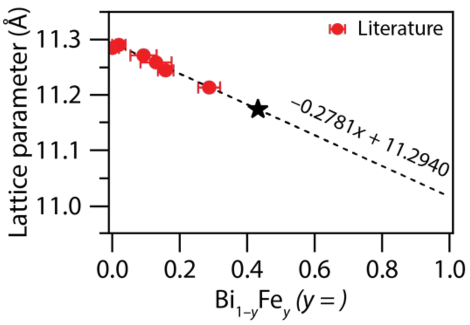

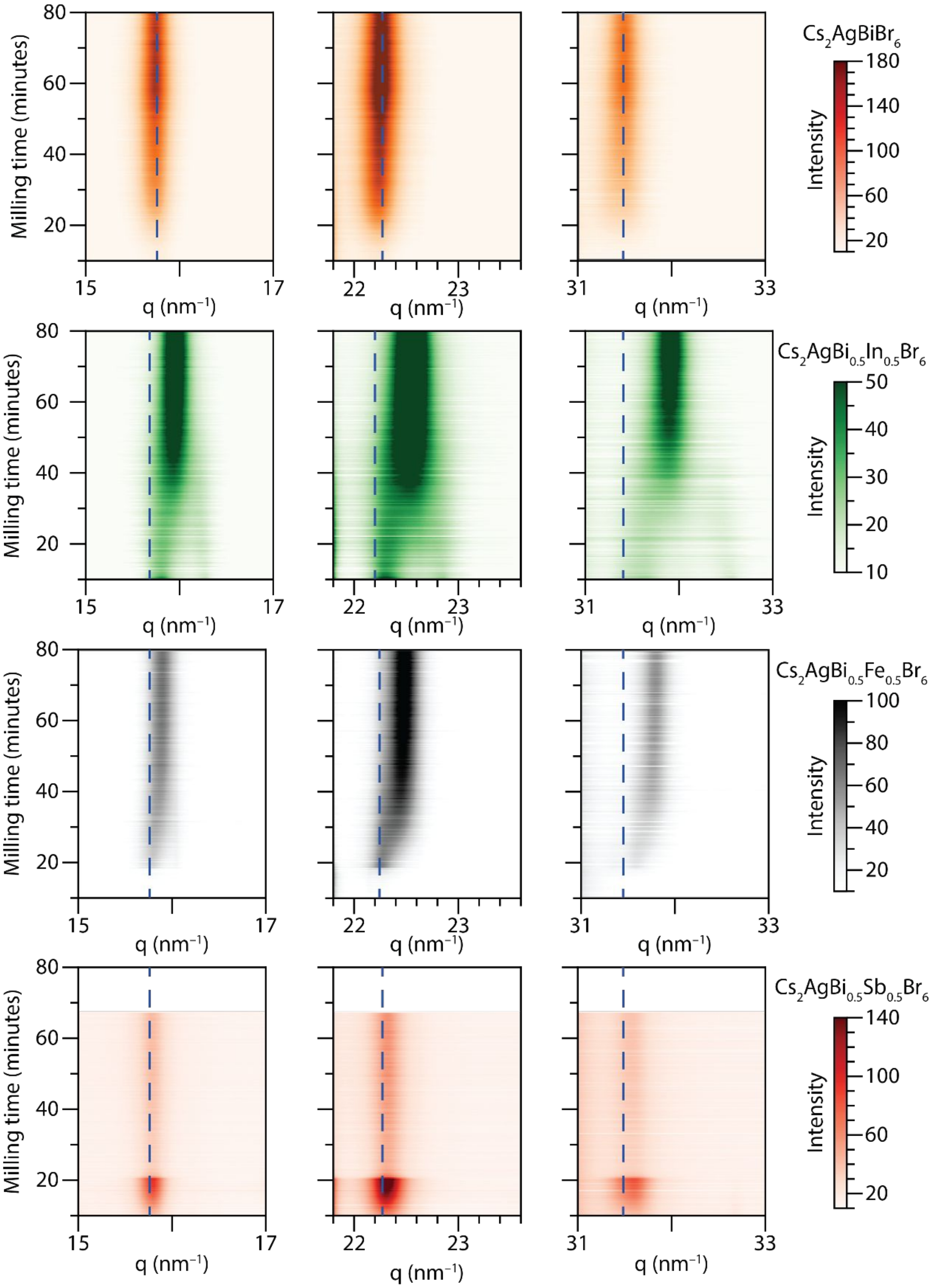

Figure S10 — Contour plot of the XRD patterns at 15–17, 21.8–23.6, and 31–33 nm^{-1} for $Cs_2AgBi_{1-y}M_yBr_6$. The rows from top to bottom correspond to $Cs_2AgBiBr_6$, $Cs_2AgBi_{0.5}In_{0.5}Br_6$, $Cs_2AgBi_{0.5}Fe_{0.5}Br_6$, and $Cs_2AgBi_{0.5}Sb_{0.5}Br_6$.

Refinement method of alloyed AgBi-elpasolites. To analyze the *in-situ* XRD patterns recorded for the synthesis of alloyed $Cs_2AgBi_{1-x}M_xBr_6$ compositions, a similar refinement procedure was used as discussed above. Each diffractogram is described using a theoretical line profile with contributions from the CsBr, $BiBr_3$, AgBr, $CsBi_2Br_7$, $Cs_3Bi_2Br_9$ and $Cs_2AgBiBr_6$ phases. The additional 7th phase, MBr_3 (M = Sb^{3+}, In^{3+} and Fe^{3+}), was not included in the refinement iterations as it does not significantly improve the fits (see **Figures S11–S13**).

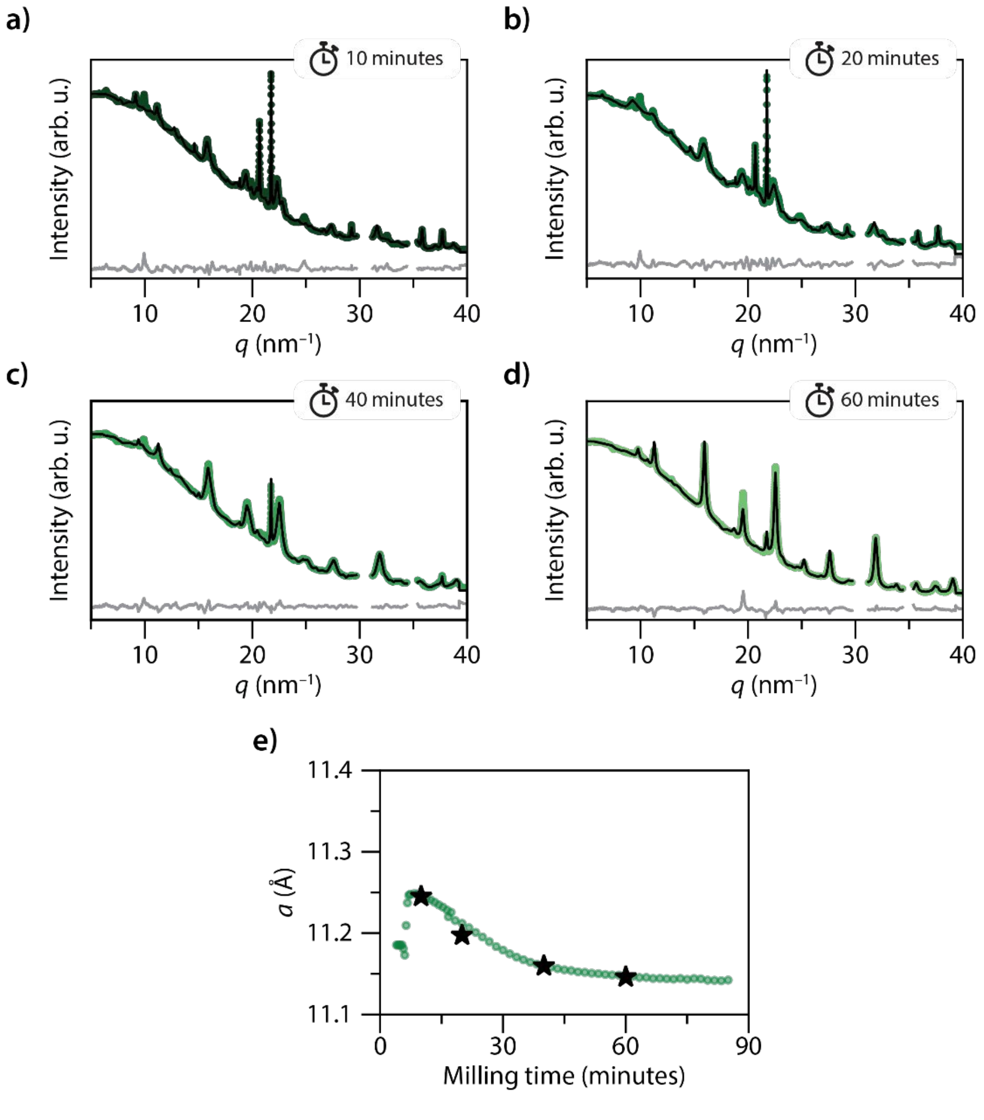

Figure S11 — Rietveld refinement $Cs_2AgBi_{0.5}In_{0.5}Br_6$. Experimental XRD pattern (green), refinement fit (black) and corresponding residual (grey) after **a)** 10, **b)** 20, **c)** 40, and **d)** 60 minutes of ball milling. **e)** The lattice parameter of $Cs_2AgBi_{0.5}In_{0.5}Br_6$ as a function of milling time. The marks correspond to the refinement fits shown in **a-d)**.

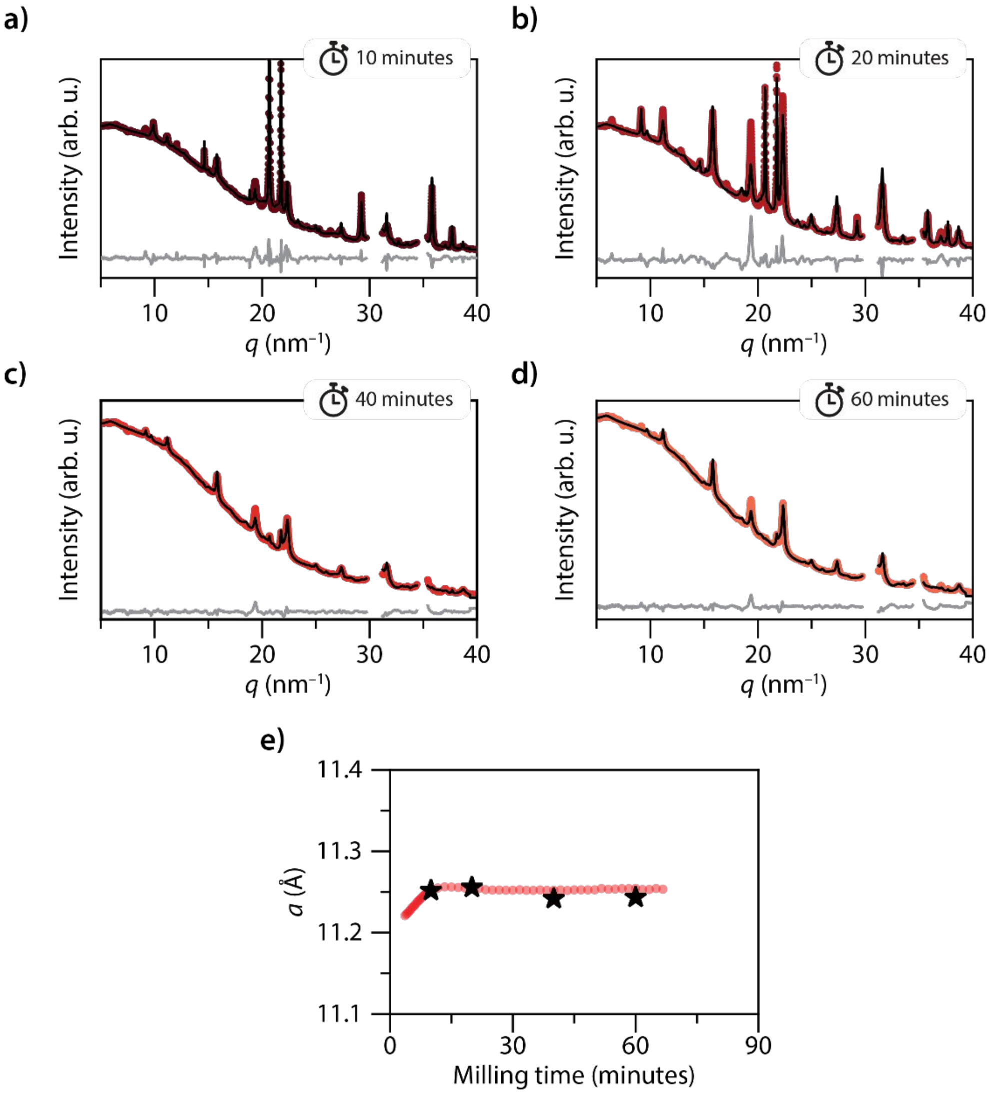

Figure S12 — Rietveld refinement $Cs_2AgBi_{0.5}Sb_{0.5}Br_6$. Experimental XRD pattern (red), refinement fit (black) and corresponding residual (grey) after **a)** 10, **b)** 20, **c)** 40, and **d)** 60 minutes of ball milling. **e)** The lattice parameter of $Cs_2AgBi_{0.5}Sb_{0.5}Br_6$ as a function of milling time. The marks correspond to the refinement fits shown in **a-d)**.

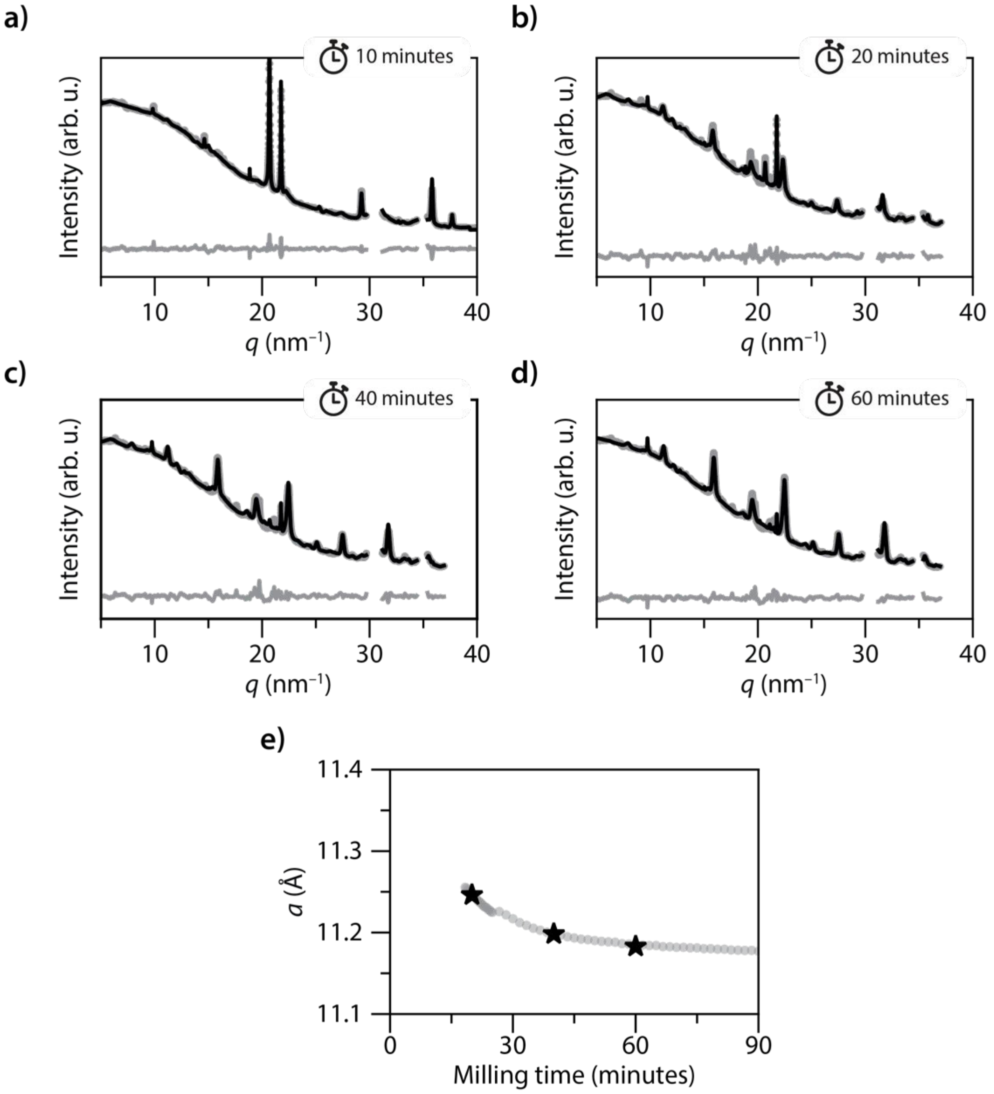

Figure S13 — Rietveld refinement $Cs_2AgBi_{0.5}Fe_{0.5}Br_6$. Experimental XRD pattern (blue), refinement fit (black) and corresponding residual (grey) after **a)** 10, **b)** 20, **c)** 40, and **d)** 60 minutes of ball milling. **e)** The lattice parameter of $Cs_2AgBi_{0.5}Fe_{0.5}Br_6$ as a function of milling time. The marks correspond to the refinement fits shown in **a-d)**.

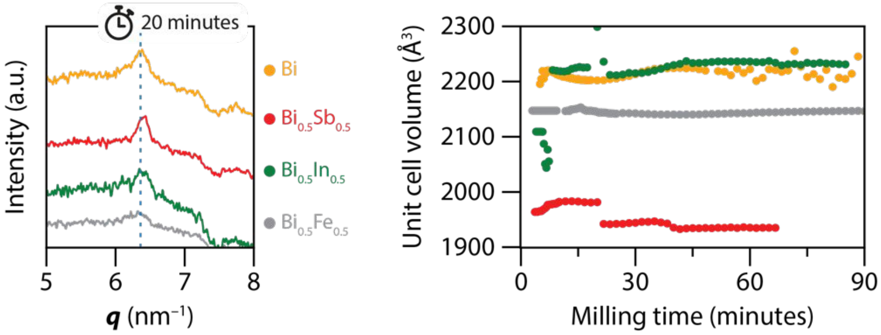

Figure S14 — *Left*: (001)-reflection of $Cs_3Bi_2Br_9$ phase. The grey dotted line serves as a guide to the eye. *Right*: Unit cell volume of the 329-phase over time.

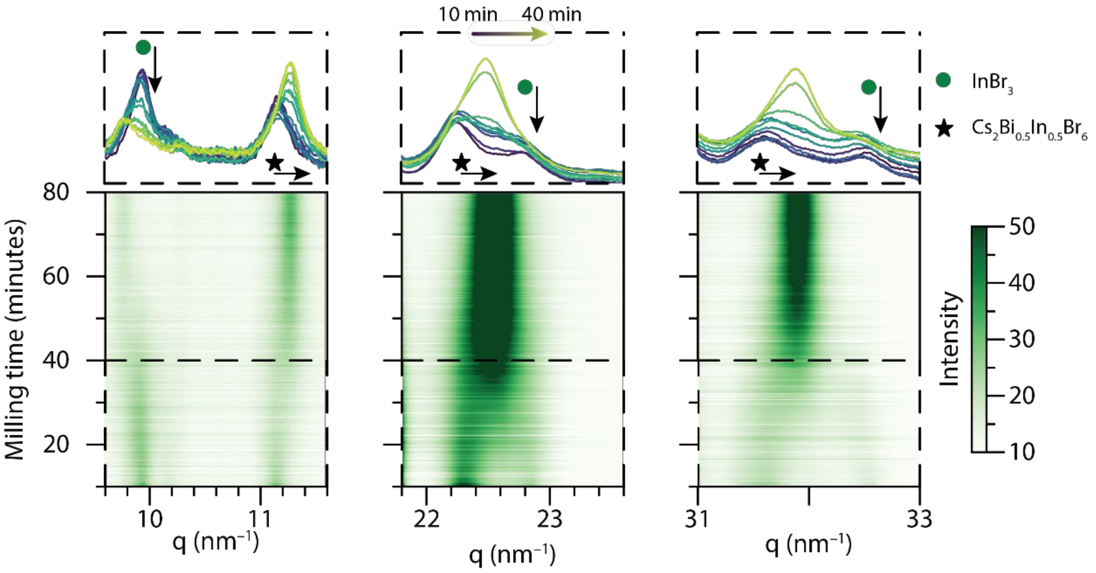

Figure S15 — Reflection intensity of $InBr_3$ vs. $Cs_2AgBi_{0.5}In_{0.5}Br_6$. Heat maps and several XRD patterns displaying the most intense reflections of $InBr_3$ (10.0 nm^{-1} (*001*-plane), 22.9 nm^{-1} (*131*-plane) and 32.69 nm^{-1} (*202*-plane)) (green dots) and the shifting elpasolite reflections (black stars).

Reference

(1)Ban, V.; Sadikin, Y.; Lange, M.; Tumanov, N.; Filinchuk, Y.; Černý, R.; Casati, N. Innovative in Situ Ball Mill for X-Ray Diffraction. *Anal. Chem.* **2017**, *89* (24), 13176–13181. https://doi.org/10.1021/acs.analchem.7b02871.

(2)Prescher, C.; Prakapenka, V. B. DIOPTAS: A Program for Reduction of Two-Dimensional X-Ray Diffraction Data and Data Exploration. *High Press. Res.* **2015**, *35* (3), 223–230. https://doi.org/10.1080/08957959.2015.1059835.

(3)Taylor, J. C.; Matulis, C. E. Absorption Contrast Effects in the Quantitative XRD Analysis of Powders by Full Multiphase Profile Refinement. *J. Appl. Crystallogr.* **1991**, *24* (1), 14–17. https://doi.org/10.1107/S002188989000841X.

(4)McCusker, L. B.; Von Dreele, R. B.; Cox, D. E.; Louër, D.; Scardi, P. Rietveld Refinement Guidelines. *J. Appl. Crystallogr.* **1999**, *32* (1), 36–50. https://doi.org/10.1107/S0021889898009856.

(5)Tagliente, M. A.; Massaro, M. Strain-Driven (0 0 2) Preferred Orientation of ZnO Nanoparticles in Ion-Implanted Silica. *Nucl. Instrum. Methods Phys. Res. Sect. B Beam Interact. Mater. At.* **2008**, *266* (7), 1055–1061. https://doi.org/10.1016/j.nimb.2008.02.036.

(6)Himabindu, B.; Latha Devi, N. S. M. P.; Rajini Kanth, B. Microstructural Parameters from X-Ray Peak Profile Analysis by Williamson-Hall Models; A Review. *Mater. Today Proc.* **2021**, *47*, 4891–4896. https://doi.org/10.1016/j.matpr.2021.06.256.

(7)Jöbsis, H. J.; Fykouras, K.; Reinders, J. W. C.; van Katwijk, J.; Dorresteijn, J. M.; Arens, T.; Vollmer, I.; Muscarella, L. A.; Leppert, L.; Hutter, E. M. Conduction Band Tuning by Controlled Alloying of Fe into $Cs_2AgBiBr_6$ Double Perovskite Powders. *Adv. Funct. Mater.* **2023**, *34*, 2306106. https://doi.org/10.1002/adfm.202306106.
